# Inhibited effects of CAPE-*p*NO_2_ on cervical carcinoma *in vivo* and *in vitro* and its detected metabolites

**DOI:** 10.18632/oncotarget.21617

**Published:** 2017-10-07

**Authors:** Xiaofang Yao, Hao Tang, Qiao Ren, Xiaoyan Zhao, Hua Zuo, Zhubo Li

**Affiliations:** ^1^ College of Pharmaceutical Sciences, Southwest University, Chongqing 400716, China; ^2^ International Academy of Targeted Therapeutics and Innovation, Chongqing University of Arts and Sciences, Chongqing 402160, China

**Keywords:** caffeic acid *p*-nitro phenethyl ester (CAPE-*p*NO_2_), cervical cancer cells, endogenous apoptosis pathway, xenograft, metabolites

## Abstract

The development of advanced cervical cancer therapies is a particularly urgent need due to the strong side effects and toxicities of current treatments. Caffeic acid phenethyl ester (CAPE) exhibits broad-spectrum antitumor activities and little toxicity or side effects. In our previous study, caffeic acid para-nitro phenethyl ester (CAPE-*p*NO_2_) significantly improved the effect of anti-platelet aggregation and attenuated myocardial ischemia. Based on this finding, we aimed to further explore the antitumor activity of CAPE-*p*NO_2_ in cervical cancer cells and tumor xenografts. In addition, we assessed the biotransformation of CAPE-*p*NO_2_ in cervical cancer cells. Our study demonstrated that both CAPE and CAPE-*p*NO_2_ can inhibit cell proliferation via the induction of G2/M cell cycle arrest. More importantly, CAPE-*p*NO_2_ dramatically induced cell apoptosis via significant down-regulation of pro-caspase-3, pro-caspase-9, Bcl-2, Cyclin B1 and Cdc2 and up-regulation of cleaved-caspase-3, Bax, CytoC and P21^Cip1^. Moreover, CAPE and CAPE-*p*NO_2_ significantly suppressed the growth and angiogenesis of nude mice xenografts. CAPE and CAPE-*p*NO_2_ were found to degrade into four and six metabolites, respectively. The metabolites of CAPE and CAPE-*p*NO_2_ were different, and the major metabolic pathway may be phase II reactions. These results suggest that CAPE-*p*NO_2_ induced cell apoptosis and cell cycle arrest via a strong regulatory effect on relevant apoptotic proteins. Therefore, CAPE-*p*NO_2_ should be further studied as a potent anti-cancer agent.

## INTRODUCTION

Cervical cancer is the third most common genital malignant tumor in women and the fourth leading cause of cancer-related death all over the world [1]. It has been reported that the prevalence of cervical cancer in China was slightly higher than in other countries. The number of cervical cancer patients is increasing at a rate of 130,000 people per year [2, 3]. At present, surgery, radiation and chemotherapy are the main treatments for cervical cancer. Unfortunately, surgery is the only standard treatment for very early-stage cervical cancer. Hence, radiation and classical chemotherapy become the backbone of cervical cancer treatment. Although combining chemotherapy with radiation has been broadly introduced as the new standard of treatment for early-stage patients, it is still limited by the high-risk of recurrence and by locally advanced disease. Moreover, chemoradiation resulted in higher acute toxicities. Therefore, it is an urgent task to develop alternative anti-cervical cancer drugs with high efficiency and low toxicity.

Apoptosis is a form of programmed cell death that is under tight genetic control, and its progress is regulated by different kinds of genes [4, 5]. The morphological changes of apoptosis, including chromatin condensation and margination, the appearance of vacuoles on the cytomembrane and the formation of apoptotic bodies, have been demonstrated [6, 7]. The mechanisms of apoptosis such as activated apoptosis signaling pathways, the mitochondria and inhibited nuclear factors have been reported [8, 9]. In addition, some apoptosis is caused by the intrinsic pathway [10–12]. In the intrinsic pathway is a pro-apoptosis gene (cytochrome C) that is released from the mitochondrial membrane into the post-cytoplasmic caspase cascade and is mainly regulated by the members of the Bcl-2 protein family. It includes anti-apoptosis genes (Bcl-2, Mcl-1, Bcl-xl) and pro-apoptosis genes (Bax, Bcl-xs, Bad) [13]. The caspases exist in the form of zymogen in the cells, they are cleaved to be in their active form by the apoptosome, eventually leading to cell structure damage or rupture and DNA damage, ultimately causing cell death [14].

Recently, the development of an antineoplastic agent has drawn considerable research attention in cancer therapy. Many cutting-edge studies have demonstrated that caffeic acid phenethyl ester (CAPE), a bioactive natural ingredient extracted from *propolis*, possesses inhibitory activity in a variety of cancers, such as cervical cancer, breast cancer, colon cancer and glioma [15–18]. CAPE could activate the intracellular and extracellular caspase apoptosis pathways as well as block survival signaling [19–21]. In addition, the expression of cell cycle arrest genes could be inhibited by CAPE [22, 23]. Our group developed a novel caffeic acid para-nitro phenethyl ester (CAPE-*p*NO_2_) that was proven to be significantly more effective than CAPE on acute myocardial ischemic and reperfusion injury in rats and other blood related diseases in previous studies [24–26]. However, the changes in structure-anticancer activity due to the introduction of the nitro-group in CAPE are still unknown. Hence, we comparatively investigate the anti-cervical cancer effects and the possible mechanisms of CAPE-*p*NO_2_ and CAPE in HeLa cells and in nude mice xenograft by detecting their metabolites with LC-QTOF-MS/MS.

## RESULTS

### CAPE and CAPE-*p*NO_2_ inhibit cell proliferation in cervical cancer cells

**Figure 1:**
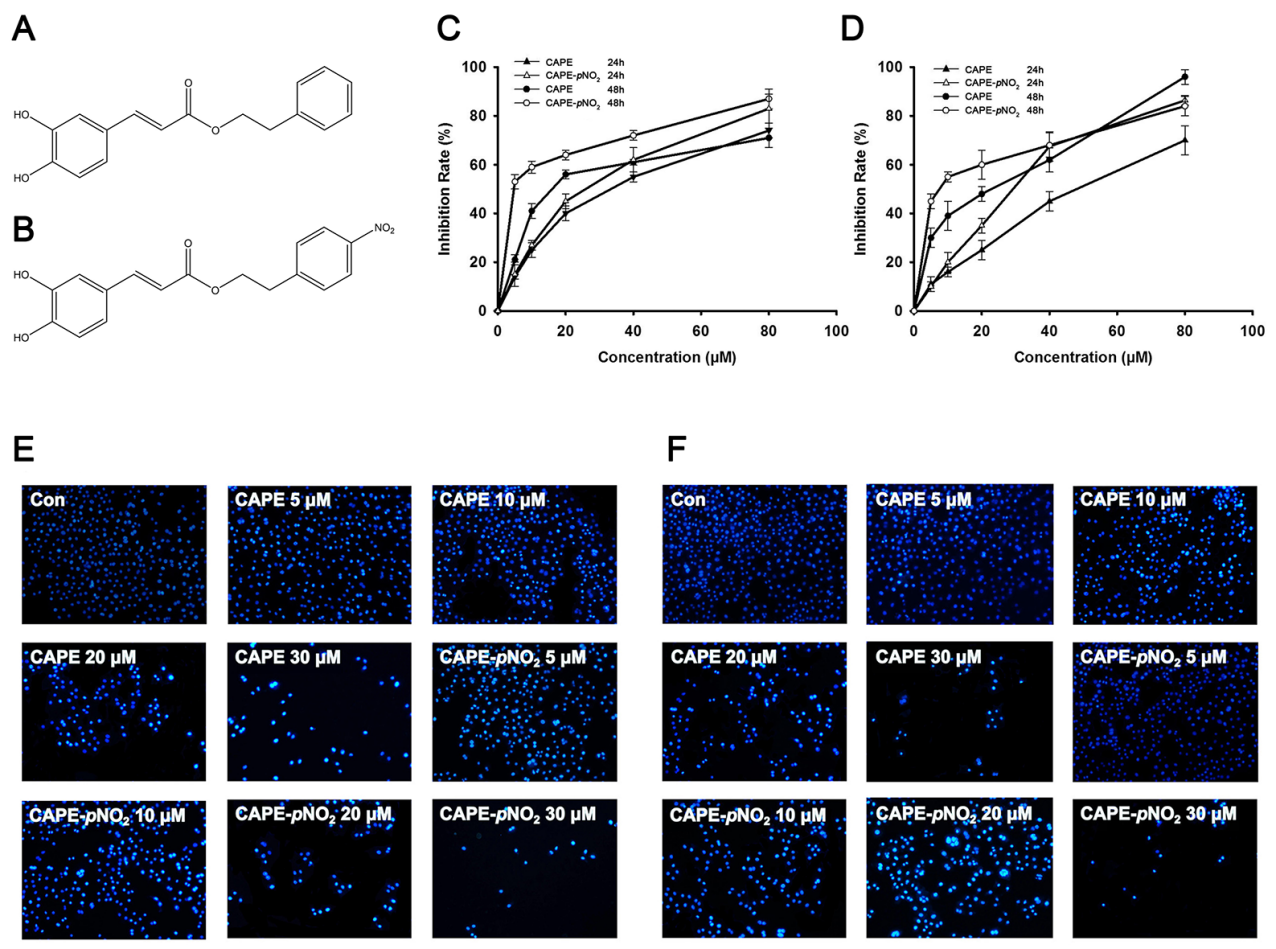
The chemical structure of CAPE and CAPE-*p*NO_2_ and results of the MTT assay and Hoechst 33342 staining **(A)** The chemical structure of CAPE. **(B)** The chemical structure of CAPE-*p*NO_2_. **(C)** HeLa cells and **(D)** Siha cells were treated with different concentrations of CAPE and CAPE-*p*NO_2_ for 24 h or 48 h and the MTT assay was performed to evaluate cell viability. **(E)** HeLa cells and **(F)** Siha cells (×400) were stained by Hoechst 33342 after treatment with different concentrations of CAPE and CAPE-*p*NO_2_ for 48 h.

The proliferation of cervical cancer cells treated with CAPE (Figure 1A) and CAPE-*p*NO_2_ (Figure 1B) was evaluated by the MTT assay. As shown in Figure 1C and 1D, the cell viability in HeLa cells and Siha cells was significantly suppressed by CAPE and CAPE-*p*NO_2_ treatment for 24 h or 48 h and showed a dose-dependent response. The IC_50_ values of CAPE and CAPE-*p*NO_2_ for 24 h were 30.3 μM and 23.5 μM, respectively, and the values for 48 h were 20.0 μM and 5.07 μM. Siha cells were treated with the same concentrations of CAPE and CAPE-*p*NO_2_ for 24 h or 48 h; the IC_50_ for CAPE was from 44.0 μM (24 h) to 23.0 μM (48 h), whereas the IC_50_ of CAPE-*p*NO_2_ was from 28.0 μM (24 h) to 7.80 μM (48 h). These results revealed that CAPE-*p*NO_2_ enhanced the anti-proliferative effect compared with CAPE. Meanwhile, HeLa cells were more sensitive to CAPE and CAPE-*p*NO_2_ than Siha cells, so HeLa cells were selected for further investigation.

### CAPE and CAPE-*p*NO_2_ induce apoptosis and necrosis in cervical cancer cells

In the present study, fluorescence microscopy measurement and flow cytometry analysis were used to confirm the apoptosis and necrosis induced by CAPE and CAPE-*p*NO_2_. Hoechst 33342 staining is shown in Figure 1E and 1F; cells in the control group were of regular roundness, the nuclear membrane was intact, and the fluorescence was homogenous. Compared with the control group, the number of cells in the treatment groups decreased significantly, and the nucleolus and chromatin shrinkage produced increased fluorescence. We compared the sensitivity between the CAPE and CAPE-*p*NO_2_ treatment groups, and the CAPE-*p*NO_2_ group palpably induced cell apoptosis.

**Figure 2:**
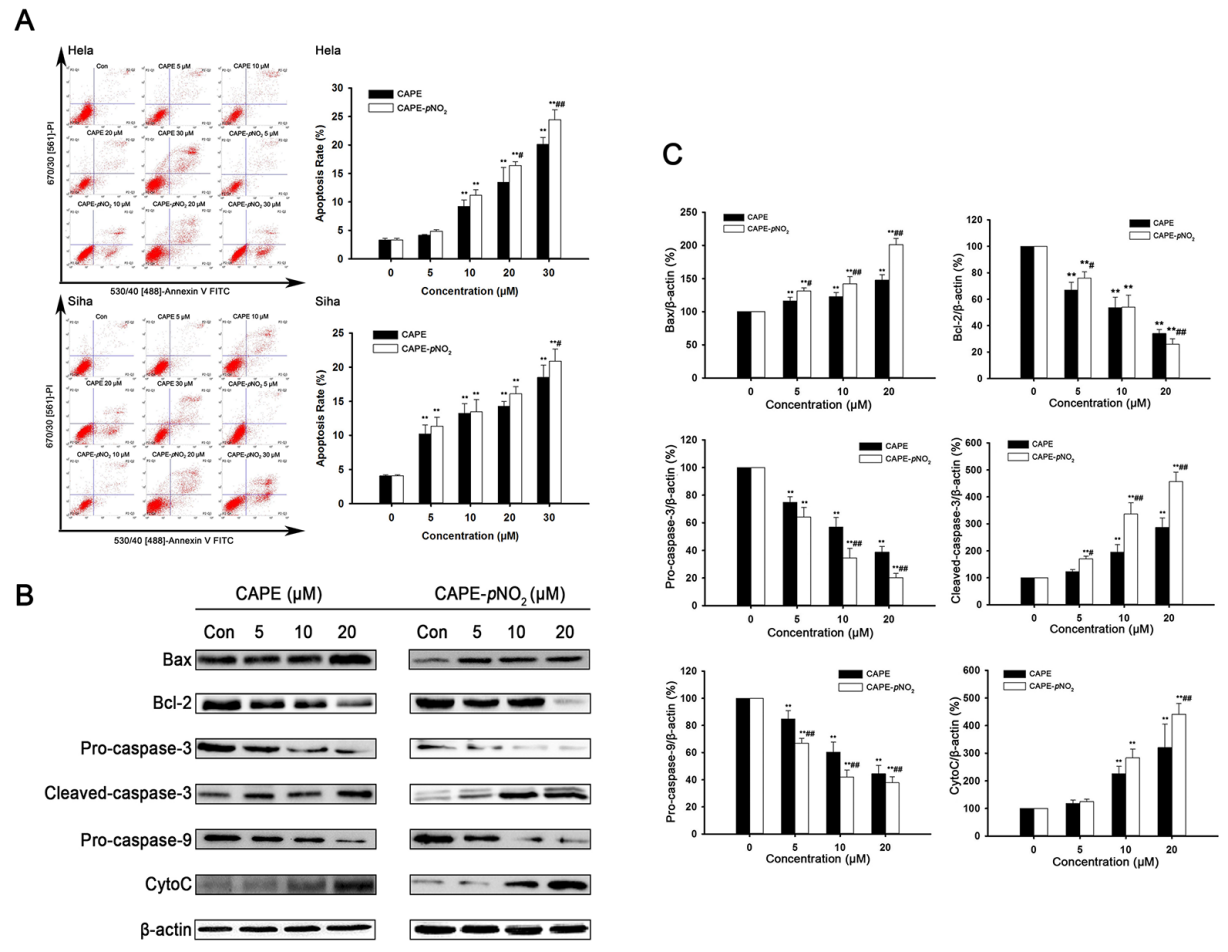
CAPE and CAPE-*p*NO_2_ induced apoptosis in cervical cancer cells **(A)** Flow cytometry analysis in cervical cancer cells. The data showed that CAPE and CAPE-*p*NO_2_ significantly induced apoptosis and necrosis in cervical cancer cells. **(B and C)** Bax, Bcl-2, CytoC, pro-caspase-9, pro-caspase-3, and cleaved caspase-3 were assessed by western blot analysis in HeLa cells.

Flow cytometry analysis demonstrated that CAPE increased apoptotic cell death in HeLa cells from 4.14 % to 20.1 %, whereas CAPE-*p*NO_2_ induced the apoptotic rate from 4.83 % to 24.5 %. Similarly, when Siha cells were treated with 30 μM CAPE or CAPE-*p*NO_2_, the apoptotic rate was 18.5 % or 20.9 % (Figure 2A), respectively. The result revealed that CAPE and CAPE-*p*NO_2_ significantly induced cell apoptosis in a dose-dependent manner. Moreover, CAPE-*p*NO_2_ had a more potent apoptotic effect than CAPE.

### CAPE and CAPE-*p*NO_2_ induce the changes in the expression of apoptotic proteins in cervical cancer cells and in tumors

**Figure 3:**
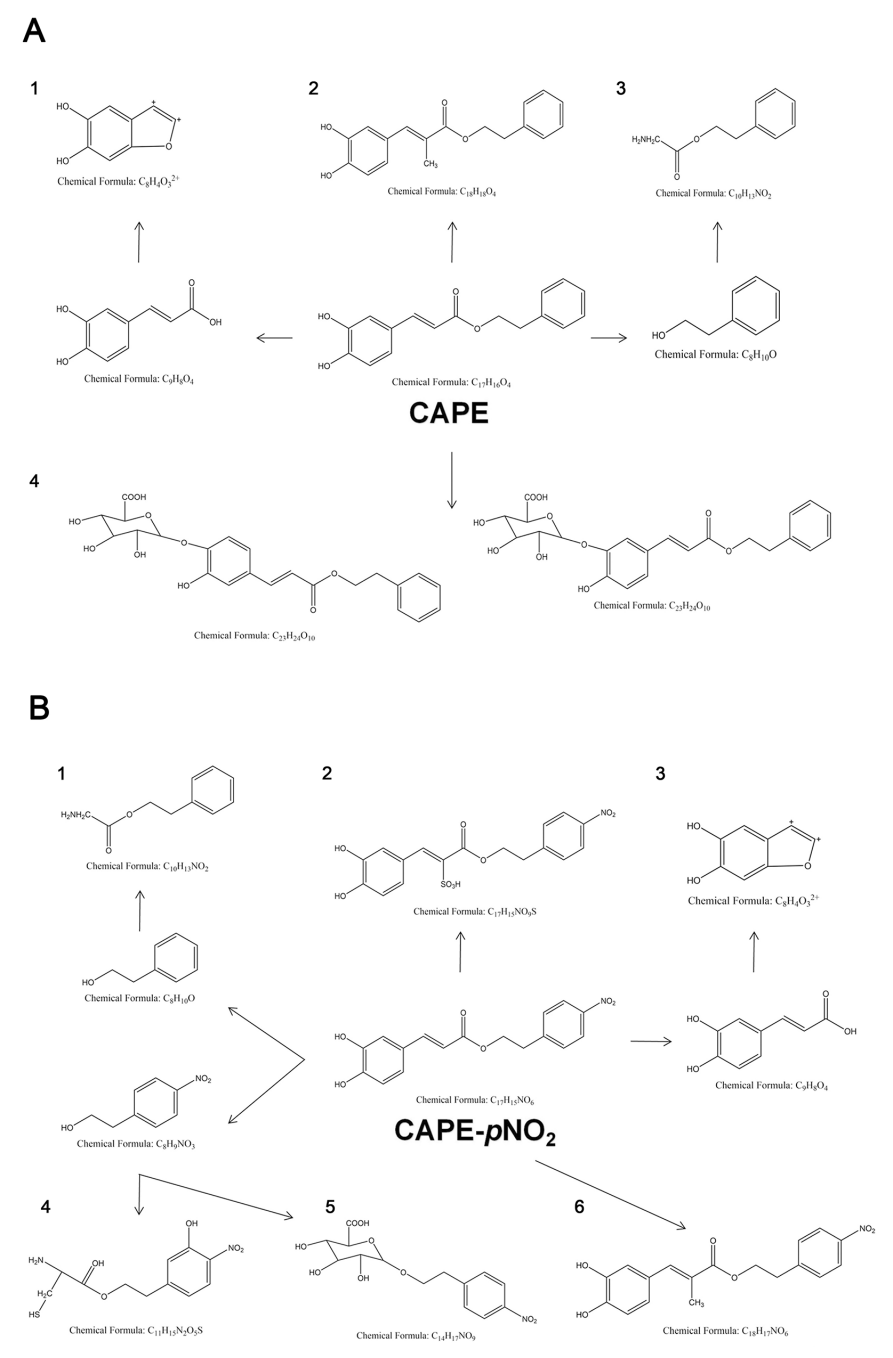
The regulation of proteins in tumors The expressions of Bax, Bcl-2, pro-caspase-3, cleaved caspase-3, cleaved caspase-9, CytoC, P21^Cip1^, Cdc2 and Cyclin B1 were detected by western blot.

Based on the previous data, HeLa cells were used to investigate the expression of Bax, Bcl-2, cleaved caspase-3, pro-caspase-3, pro-caspase-9 and CytoC induced by CAPE and CAPE-*p*NO_2_. Compared with the control group, CAPE and CAPE-*p*NO_2_ treatment significantly increased the protein levels of Bax, CytoC and cleaved caspase-3, whereas these treatments significantly decreased the levels of Bcl-2, pro-caspase-3 and pro-caspase-9 (Figure 2B, 2C). After treating with 20 μM CAPE and CAPE-*p*NO_2_, the expression of Bax was up-regulated 1.47 and 2.10 times, respectively, and Bcl-2 was down-regulated 2.92 and 3.83 multiples, respectively. Meanwhile, the expression of cleaved caspase-3 was up-regulated 2.87 and 4.57 times after 20 μM CAPE and CAPE-*p*NO_2_ treatment. In tumors, CAPE and CAPE-*p*NO_2_ up-regulated Bax, CytoC, cleaved-caspase-3 and cleaved-caspase-9, and down-regulated pro-caspase-3 and Bcl-2; moreover, CAPE-*p*NO_2_ regulation of these proteins was in a dose-dependent manner (Figure 3). These results suggested that CAPE-*p*NO_2_ was more effective than CAPE in the induction of cell apoptosis.

### CAPE and CAPE-*p*NO_2_ induce G2/M-phase arrest in HeLa cells

**Figure 4:**
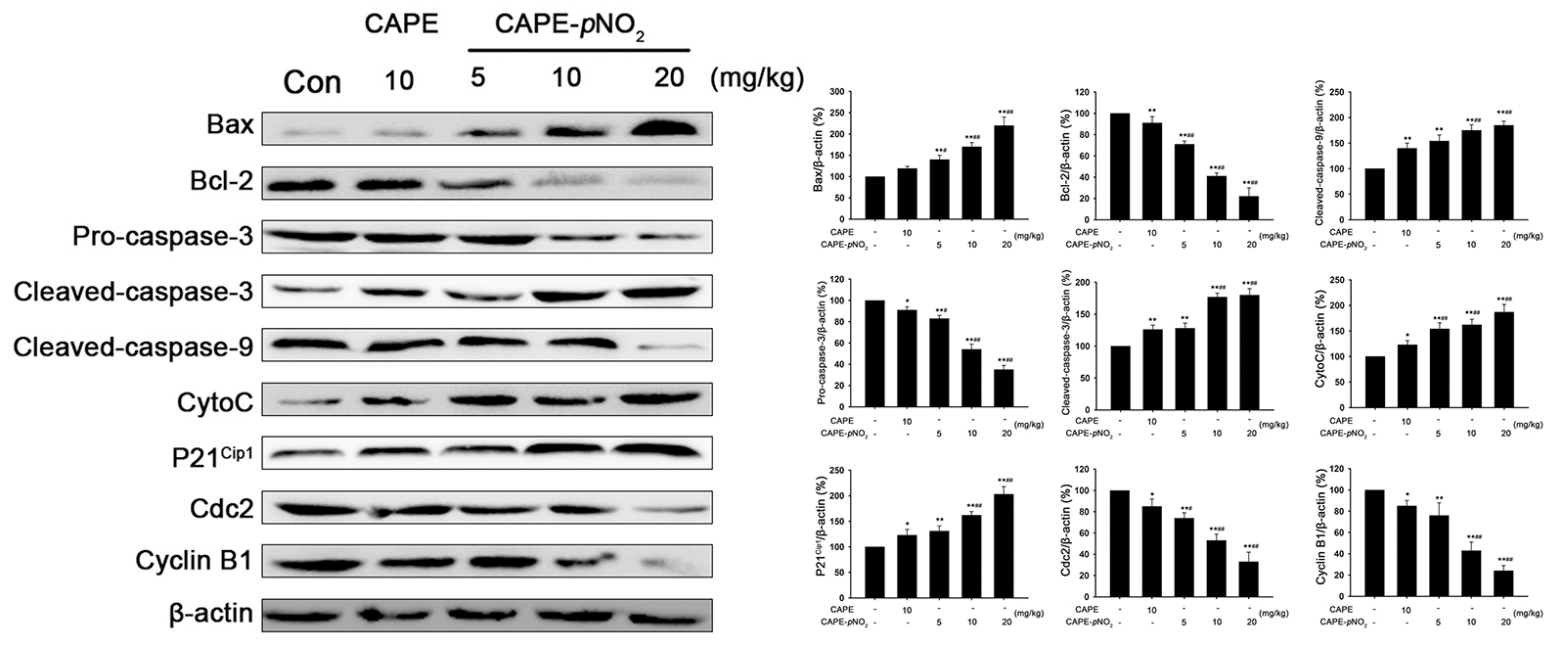
CAPE and CAPE-*p*NO_2_ induced G2/M-phase arrests in HeLa cells **(A)** PI staining in HeLa cells. **(B and C)** The cell cycle related proteins P21^Cip1^, Cdc2, Cyclin B1 were assessed by western blot analysis.

To evaluate the effects of CAPE and CAPE-*p*NO_2_ on cell cycle distribution, flow cytometry was used. As shown in Figure 4A, the number of HeLa cells in G2/M phase was notably increased from 4.51 % to 11.9 % after treatment with CAPE for 48 h. However, the data showed that the amount of HeLa cells in G2/M phase increased markedly from 6.90 % to 26.9 % in the CAPE-*p*NO_2_ treatment group.

Further results showed that cell cycle progression is tightly regulated by the expression of P21^Cip1^, Cdc2 and Cyclin B1. As shown in Figure 4B and 4C, drug treatment increased the protein levels of P21^Cip1^ and decreased the expression of Cyclin B1 and Cdc2. After treatment with 20 μM CAPE-*p*NO_2_, the expressions of Cyclin B1 and Cdc2 were down-regulated 0.37 and 0.33 times, respectively, in comparison with the control group. With increased concentrations of CAPE treatment, the level of Cyclin B1 and Cdc2 increased 0.5 and 0.48 multiples, respectively. These results suggested that CAPE and CAPE-*p*NO_2_ caused G2/M cell cycle arrest in HeLa cells in a significantly dose-dependent manner. In tumors, CAPE and CAPE-*p*NO_2_ increased the expression of P21^Cip1^, decreased the expression of Cdc2 and Cyclin B1. The effect of CAPE-*p*NO_2_ on proteins was in a dose-dependent manner (Figure 3). Furthermore, CAPE-*p*NO_2_ has more effective than CAPE.

### CAPE and CAPE-*p*NO_2_ cause retardation of xenograft in nude mice

**Figure 5:**
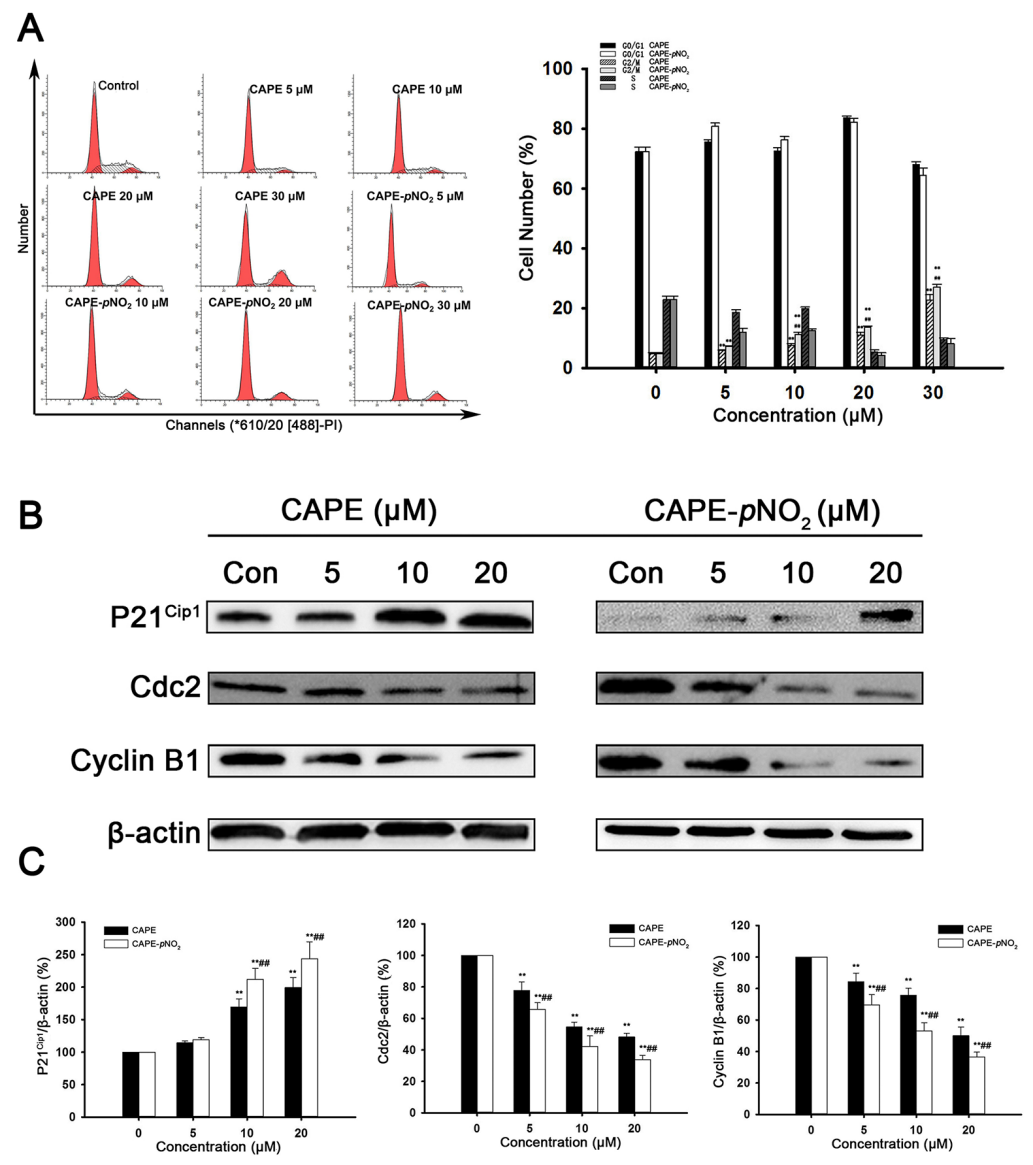
CAPE and CAPE-*p*NO_2_ caused the retardation of xenografts in nude mice **(A and B)** HeLa cells were implanted subcutaneously into nude mice. CAPE (10 mg/kg/day), CAPE-*p*NO_2_ (5, 10, 20 mg/kg/day) or vehicle was administered by intragastric administration. The tumor volume in mice carrying HeLa xenografts was measured every 3 days. **(C and D)** Tumors were excised and weighed on day 41. **(E)** H9c2 cells were treated with increasing concentrations of CAPE and CAPE-*p*NO_2_ for 48 h, and the MTT assay was used to evaluate cell proliferation.

To verify the effects of CAPE and CAPE-*p*NO_2_ on tumor growth, HeLa cells were injected into nude mice. As shown in Figure 5A and 5B, after administration of CAPE and CAPE-*p*NO_2_ by gavages for 41 days, CAPE (10 mg/kg/day) and CAPE-*p*NO_2_ (5 mg/kg/day, 10 mg/kg/day, 20 mg/kg/day) exhibited a 44.9 %, 53.7 %, 59.9 % and 67.2 % inhibition on tumor growth, respectively. The tumor weight showed a significant decrease in mice treated with CAPE and CAPE-*p*NO_2_ compared to the control group mice (Figure 5C and 5D). Morphological changes were not found in the heart, liver, spleen and kidney cells, and all groups demonstrated no overt difference in body weight of the mice (Supplementary Figure 1). The MTT assay was used to detect the viability of H9c2 cells after treatment with CAPE and CAPE-*p*NO_2_. The results suggested that CAPE and CAPE-*p*NO_2_ were not overtly toxic (Figure 5E).

### CAPE and CAPE-*p*NO_2_ induce more apoptosis or necrosis in xenograft tumors

**Figure 6:**
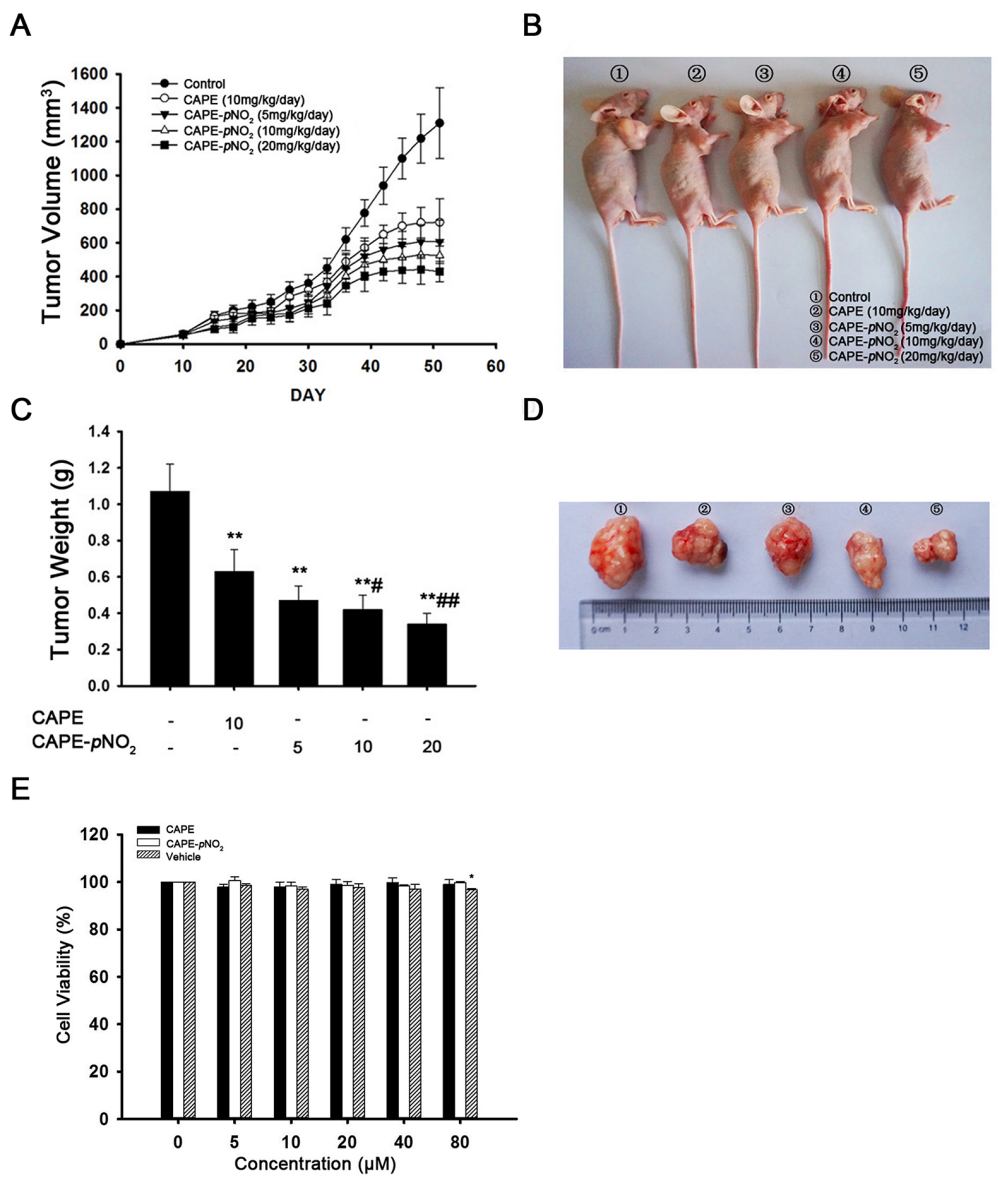
HE staining, TUNEL staining and immunohistochemical assessment of the xenograft tumors **(A)** HE staining. **(B)** TUNEL assay. **(C)** The tumor vascularity was detected by immunofluorescent staining of VEGF.

Histopathological and immunohistochemical staining was used to evaluate whether the CAPE and CAPE-*p*NO_2_ treatment induced more apoptosis or necrosis in the xenografts of HeLa cells. The results of HE staining revealed that the structure of the tumor in the control group was clear and compact, but in the CAPE and CAPE-*p*NO_2_ treatment group, we could clearly observe early changes of cellular necrosis under the electron microscope; for example, the nuclear membrane had some shrinkage, and the cells turned smaller and more round (Figure 6A).

It is well known that genomic DNA during apoptosis may yield some DNA fragments, which can be identified by labeling free the 3′-OH termini with modified nucleotides in a template-independent manner (TUNEL reaction). Compared with the control group, the number of positive cells increased significantly. The statistical analysis of the pathological tissue sections demonstrated that the percentage of positive cells was 1.8 %, 2.1 %, 3.2 %, and 4.5 % by CAPE and CAPE-*p*NO_2_ treatment. The results indicated that CAPE and CAPE-*p*NO_2_ can up-regulate the tumor apoptosis rate (Figure 6B).

### CAPE and CAPE-*p*NO_2_ inhibit tumor vascularity

Immunohistochemical staining of VEGF was carried out to detect tumor vascularity. VEGF is a major factor in the regulation of angiogenesis. As shown in Figure 6C, the expression level of VEGF was lower in the CAPE-*p*NO_2_ group than in the control and CAPE group. In addition, the concentration was negatively correlated with the expression of VEGF in the CAPE-*p*NO_2_ group, which indicated that CAPE-*p*NO_2_ possessed a strong inhibitory effect on tumor vascularity.

### The metabolism of CAPE and CAPE-*p*NO_2_ in HeLa cells

**Figure 7:**
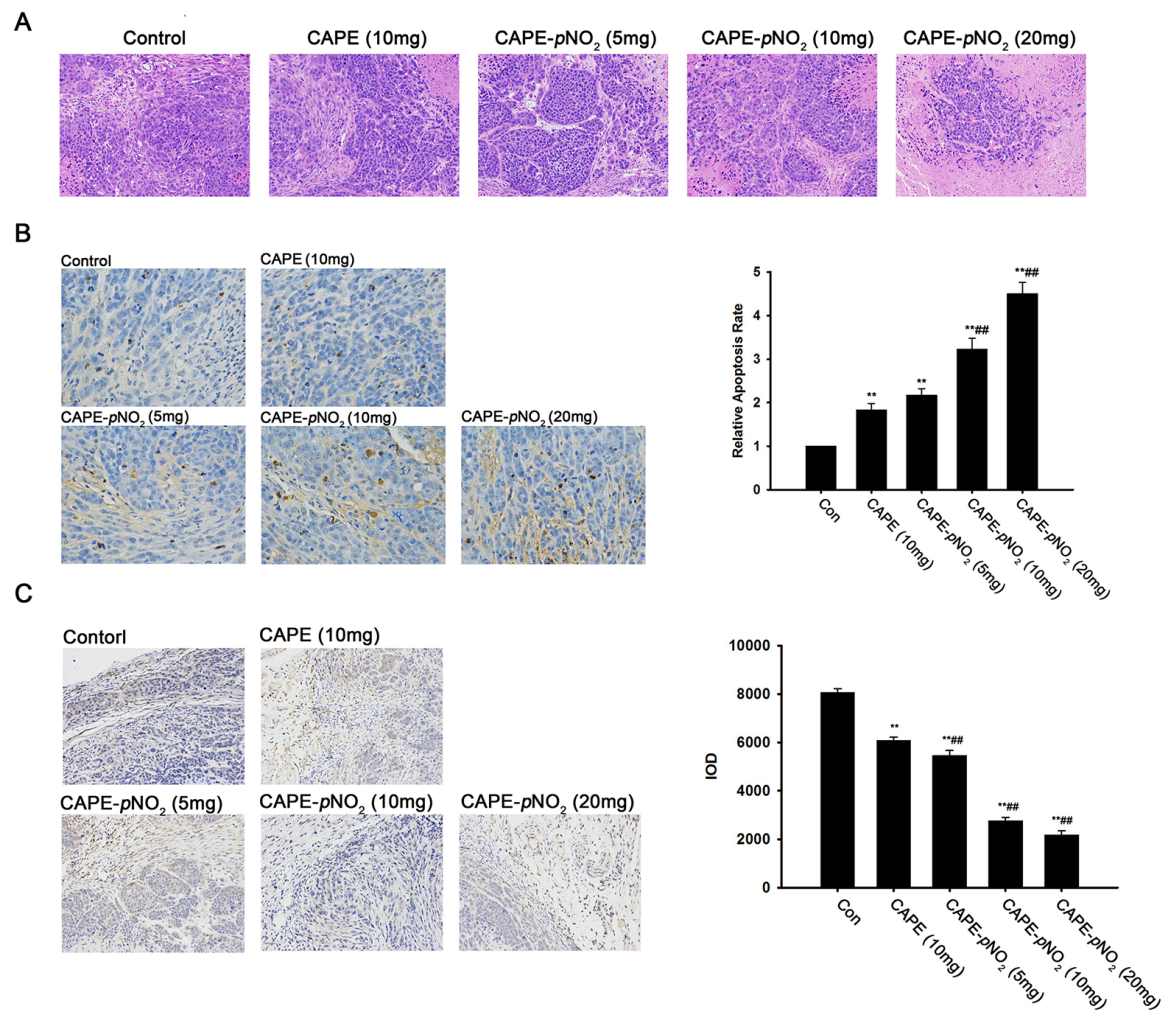
The metabolic process of CAPE and CAPE-*p*NO_2_ in HeLa cells **(A)** With the “1, 2, 3, 4” representing 5,6-dihydroxybenzofuran-2,3-diylium; phenethyl (E) -3- (3,4-dihydroxyphenyl) -2-methylacrylate; phenethyl glycinate, (2S, 3S, 4R, 5R, 6S) -3, 4,5-trihydroxy-6- (2-hydroxy-5- ((E) -3-oxo-3-phenethoxyprop-1-en-1-yl) phenoxy) tetrahydro-2H-pyran-2-carboxylic acid; and (2S, 3S, 4R, 5R, 6S)-3, 4,5-trihydroxy-6- (2-hydroxy-4-((E) -3-oxo-3-phenethoxyprop-1-en-1-yl) phenoxy) tetrahydro- 2H-pyran-2-carboxylic acid, respectively. **(B)** With the “1, 2, 3, 4, 5, 6” representing phenethyl glycinate; (Z) -1- (3,4-dihydroxyphenyl) -3- (4-nitrophenethoxy) -3-oxoprop-1-ene-2-sulfonic acid; 5,6-dihydroxybenzofuran-2,3-diylium; 5- (2- (2-amino-3-mercapto-1- (l3-oxidanylidene) propoxy)ethyl) -2-nitrophenol; (2S, 3S, 4R, 5R) -3, 4,5-trihydroxy-6-(4-nitrophenethoxy) tetrahydro-2H-pyran-2- carboxylic acid; and 4-nitrophenethyl (E) -3- (3,4-dihydroxyphenyl) -2-methylacrylate, respectively.

To study the biotransformation of CAPE and CAPE-*p*NO_2_ in HeLa cells, with the help of MetabolitePilot software and the metabolite prediction function of mass defect filter, we speculated the molecular formula of the corresponding compounds of various kinds of spectral peaks. As shown in Figure 7, CAPE and CAPE-*p*NO_2_ may have four and six metabolites, respectively. The LC/MS/MS chromatograms of the metabolites are in the supplement (Supplementary Figures 2, and 3). The studies showed that phenethyl glycinate and 5,6-dihydroxybenzofuran-2,3-diylium were both in CAPE and CAPE-*p*NO_2_. In addition, the major metabolic pathway of CAPE and CAPE-*p*NO_2_ may be a phase II reaction (biotransformed by glucuronidation, sulfonation or methylation), although a small quantity of them could be metabolized via a phase I reaction, that might be hydrolysis.

## DISCUSSION

Many studies have suggested that CAPE, as a new anti-carcinogenic agent, could induce cell apoptosis and suppress both tumor growth and cell cycle arrest [27–29]. For example, CAPE induces E2F-1-mediated growth inhibition in human cervical cancer cells [15]. CAPE and CAPE-*p*NO_2_ were synthesized in our laboratory, and the difference between them is that CAPE-*p*NO_2_ has a nitro group in the para position. CAPE-*p*NO_2_ had more significant influences than CAPE in the previous study. It is reported that para-NO-ASA can selectively induce apoptosis in primary CLL cells and efficiently reduces tumor growth in a CLL-like xenograft model [30]. However, whether CAPE-*p*NO_2_ has the same anti-cancer effect still needs to be demonstrated.

In the present study, the inhibitory effects of CAPE and CAPE-*p*NO_2_ on the proliferation of cervical cancer cells were tested. The MTT assay demonstrated that CAPE and CAPE-*p*NO_2_ treatment for 24 h or 48 h significantly inhibited the cell viability of HeLa and Siha cells, and CAPE-*p*NO_2_ is more effective than CAPE. Hoechst 33342 staining and flow cytometry analysis also demonstrated that CAPE-*p*NO_2_ induced more apoptosis than CAPE. CAPE and CAPE-*p*NO_2_ treatment has not overtly toxic in the organs of nude mice and H9c2 cells, and it is obvious that CAPE-*p*NO_2_ is a safer compound than CAPE according to the morphological changes.

When the cell is stimulated by the internal apoptosis stimulating factor, such as oncogene activation, DNA damage, cell hypoxia or cell growth factor deletion, the factor can activate the mitochondrial apoptosis pathway and induce cell apoptosis. CytoC was one in this pathway that released from the mitochondria into the cytoplasm and activated the caspase; CytoC plays a key role in the process of cell apoptosis [31–33]. The release of CytoC is regulated by the Bcl-2 family. The family of Bcl-2 includes the anti-apoptosis genes (Bcl-2, Mcl-1, Bcl-xL) and the pro-apoptosis genes (Bax, Bcl-xs, Bad) [34]. Previous studies have suggested that the Bax gene may be related to the occurrence of thyroid cancer; Bcl-2 plays a prominent role in determining the threshold of apoptotic sensitivity [35–37]. In our study, the level of CytoC was increased 4.4 times after treatment with 20 μM CAPE-*p*NO_2_, and the up-regulation of Bax and down-regulation of Bcl-2 suggested that CAPE-*p*NO_2_ induced cervical cancer cell apoptosis by affecting the expression of Bcl-2 family proteins. After CytoC enters the cytoplasm, it activates Apaf-1, and then CytoC, Apaf-1 and pro-caspase-9 form apoptotic bodies, finally activating pro-caspase-3; caspase-3 is the main effect factor in the process of cell apoptosis [38, 39]. The present study demonstrated that the level of cleaved caspase-3 was increased approximately 4.57 times and 2.87 times with 20 μM CAPE-*p*NO_2_ and CAPE, respectively; this result means that the two substances might enhance the mitochondrial apoptosis pathway. Meanwhile, the up-regulated P21^Cip1^ and down-regulated Cyclin B1 and Cdc2 in the present study implied that CAPE-*p*NO_2_ and CAPE could arrest the cell cycle in G2/M phase. All results suggested that both CAPE and CAPE-*p*NO_2_ could significantly increase related protein expression via the internal mitochondrial pathway, and the effect of CAPE-*p*NO_2_ treatment is stronger than that of CAPE.

We have further verified these results *in vivo*. Compared to the control group, the tumor growth rate began to slow down after treatment with CAPE for 35 days, and the tumor growth in all the CAPE-*p*NO_2_ (5, 10, 20 mg/kg/day) groups began to decrease in 29 days. In addition, the concentration was negatively correlated with the tumor size of the CAPE-*p*NO_2_ groups, which indicated that CAPE-*p*NO_2_ had a more inhibitory effect on tumor growth than CAPE.

Tumor growth requires abundant nutrition. Generating blood vessels for nutrients and oxygen to enter the tumor tissue for metabolism provides the basis for growth, so the formation of tumor angiogenesis plays a crucial role in tumor growth [40]. VEGF is necessary for angiogenesis [41], and the high expression of VEGF promotes the growth and metastasis of tumors [42]. The results of the immunohistochemistry assay showed that the level of VEGF was down-regulated by CAPE and CAPE-*p*NO_2_ treatment. In addition, the positive expression of VEGF in the CAPE-*p*NO_2_ group was lower than in the CAPE group, which means that CAPE-*p*NO_2_ strongly inhibited tumor growth and reduced tumor cell migration and invasion.

The present data demonstrated that CAPE and CAPE-*p*NO_2_ may have four or six metabolites, and is result is remarkably different than the result of the previous study. The study speculated that the main metabolic products of CAPE-*p*NO_2_ included caffeic acid, ferulic acid, nitro-ferulic acid, phenethyl ester and nitro-beta-phenylethyl alcohol [43]. The difference between CAPE and CAPE-*p*NO_2_ is mainly because the metabolic reaction environment *in vitro* cannot completely simulate the body’s metabolic environment and because of the differences between the species of metabolic enzymes and their expression. Our results indicate that the presumed basis of the previous metabolism studies is insufficient. In addition, the glycine in the culture medium is perhaps the cause of the formation of phenethyl glycinate.

In conclusion, our findings demonstrated that CAPE-*p*NO_2_ exhibited significantly higher anti-cervical cancer activity than CAPE, as evidenced by the inhibition of cell viability and cell cycle arrest. The anticancer effect of CAPE-*p*NO_2_ is via the endogenous apoptosis pathway both *in vitro* and *in vivo*; CAPE-*p*NO_2_ dramatically decreased the expression of pro-caspase-3, pro-caspase-9 and Bcl-2 and increased the expression of cleaved-caspase-3, Bax and CytoC. CAPE-*p*NO_2_ may have six metabolites, and the major metabolic pathway of CAPE-*p*NO_2_ may be phase II reactions. (Z)-1-(3,4-dihydroxyphenyl)-3-(4-nitrophenethoxy)-3-oxoprop-1-ene-2-sulfonic acid is a particular metabolite of CAPE-*p*NO_2_; it may be the reason CAPE-*p*NO_2_ showed improved inhibition over CAPE. Therefore, CAPE-*p*NO_2_ could be used as a hopeful and potential candidate drug for cervical cancer. Further investigation into the mechanism of CAPE-*p*NO_2_ on cervical cancer is ongoing in our laboratory.

## MATERIALS AND METHODS

### Materials

Caffeic acid phenethyl ester (CAPE) and caffeic acid para-nitro phenethyl ester (CAPE-*p*NO_2_) were synthesized by methods described in the literature [44]. Dulbecco’s modified Eagle’s medium-high glucose (DMEM-H) culture medium, fetal bovine serum (FBS), penicillin, streptomycin, dimethyl sulfoxide (DMSO), trypsin-EDTA, Hoechst 33342, albumin from bovine serum (BSA), diaminobenzidine (DAB), hematoxylin, eosin, TUNEL kit, and 3-[4,5-dimethyl-2-thiazolyl]-2,5-diphenyl-2-tetrazolium bromide (MTT) were purchased from Sigma chemicals (St. Louis, MO). HPLC grade methanol, formic acid, acetonitrile and trichloromethane were purchased from Fisher Scientific (Fair Lawn, NJ, USA). Hela, Siha and H9c2 cell lines were obtained from Shanghai Cell Collection (Shanghai, China). Primary and secondary antibodies of P21^Cip1^, pro-caspase-9, β-actin, Bax, Bcl-2, CytoC, cleaved caspase-3, pro-caspase-3, cyclin B1, Cdc2, VEGF, and secondary antibody (HRP marked) were purchased from Proteintech Group Inc. (Wuhan, China). All other reagents were of analytical grade and were used without further purification.

### Cell culture

The Hela, Siha and H9c2 cells were maintained at 37°C with 5 % CO_2_ in DMEM-H supplemented with 10 % FBS and 1 % Pen/Strep.

### Cell viability assay

The MTT assay was used to determine the effect of CAPE and CAPE-*p*NO_2_ on cell proliferation ability [27]. Hela, Siha and H9c2 cells were seeded into 96-well plates at a density of 3×10^3^ cells per well and cultured for 24 h. The cells were then treated with concentrations (0, 5, 10, 20, 40, 80 μM) of CAPE and CAPE-*p*NO_2_ for 24 h or 48 h; MTT (5 mg/mL) was added to each well and incubated at 37°C for another 4 h. The absorbance (OD) was determined by a microplate reader (Bio Tek) at 570 nm.

### Hoechst 33342 staining

Hela and Siha cells were seeded into confocal culture dishes at a density of 1×10^4^ cells per well and allowed to attach overnight. Cells were then treated with different concentrations of CAPE and CAPE-*p*NO_2_ (0, 5, 10, 20 and 30 μM) for 48 h. Then, cells were fixed with a 4 % formaldehyde solution and rinsed with PBS three times. The cells were stained with Hoechst 33342 (10 mg/mL) for 10 min and kept away from light exposure. After washing, the cells were immediately examined using a fluorescence microscope of 400× (Olympus U-RFLT50, Tokyo, Japan).

### Apoptosis analysis

Apoptosis in HeLa and Siha cells was evaluated using Annexin V-FITC/PI staining as described. Briefly, HeLa and Siha cells were cultured with various concentrations of CAPE and CAPE-*p*NO_2_ in 6-well plates for 48 h. All the attached and floating cells were collected, rinsed three times with PBS and resuspended in 500 mL of binding buffer. Then, Annexin V-FITC (5 μL) and PI (5 μL) were added, and flow cytometry was performed after 30 min.

### Cell cycle analysis

To determine cell-cycle distribution, HeLa cells were treated with different concentrations (0, 5, 10, 20, 30 μM) of CAPE and CAPE-*p*NO_2_. After 48 h, the cells were collected and fixed with 70 % cold ethanol overnight at 4°C. Then, cells were washed and stained with 50 μg/mL PI at room temperature for 30 min in the dark. Flow cytometry was performed using a FACSCalibur flow cytometer (Becton-Dickinson, USA) equipped with a 488 nm argon laser. The number of events was evaluated for each sample, and the data were analyzed using Cell Quest software and ModiFit.

### Animal experiment

Experiments involving female Balb/c nude (nu/nu) mice (4 weeks old) were approved by the Animal Center at the Beijing HFK Bioscience Co. Ltd. (Beijing China). HeLa cells (1.0×10^7^ cells/mL) were injected subcutaneously into the left foreleg of the mice. When the average size of the tumors reached 100 mm^3^, the mice were randomly separated into five groups: the CAPE group (10 mg/kg/day in sesame oil), the CAPE-*p*NO_2_ group (5, 10, 20 mg/kg/day in sesame oil) and the control group. Tumor size and weight were measured every three day using calipers. The volume of the tumor was calculated based on the following formula: length×width^2^×π/6 = volume (mm^3^) [45]. After 41 days, the mice were sacrificed according to the institutional guidelines, and the tumors were removed and weighed.

### HE staining

The organs and tumors from nude mice were fixed in 4 % polyformaldehyde for over 24 h. Tissue specimens were dehydrated by an alcohol gradient and soaked in wax before being put into the embedding machine. After embedding, organs and tumors were placed in an Ultra-Thin Semiautomatic Microtome. The paraffin sections were dewaxed in water and put into hematoxylin for 8 min. Then, sections were added to eosin for 3 min. Stained sections were dehydrated and sealed using neutral gum. After staining, the nucleus presented as blue and the cytoplasm as red by microscopy.

### Immunohistochemical staining of tumor tissues

The paraffin sections were dewaxed in water, then protease K working fluid was dropped in the circle which was marked by an immunohistochemical pen at 37°C for 30 min. Slides were covered with PBS (pH 7.4) three times on the shaking table. Sections were incubated for 30 min after tissues were covered with detergent working fluid. Then, slides were washed three times with PBS (pH 7.4), again. Reagent 1 (TdT) and reagent 2 (dUTP) in the TUNEL kit were mixed at a ratio of 2:29, the reagent mixture was added to the circle. After incubating for 30 min at 37°C, a 3 % hydrogen peroxide solution was prepared in methanol and was used for blocking the endogenous peroxidase. Each slide was covered with reagent 3 (converter-POD) for half an hour in a 37°C warm box. Sections were stained by DAB (diaminobenzidine), and the nucleus was stained by Harris hematoxylin. Last, neutral gum was used for sealing stained sections.

Tumor vascularity was determined by staining tumor sections with VEGF antibodies. Tumor tissues sections were treated with 0.3 % hydrogen peroxide to block the endogenous peroxide activity. Then, pieces were sealed with BSA for 30 min at room temperature. Next, anti-VEGF antibody covered the paraffin to incubate overnight, and then the secondary antibody marked with HRP was added for 50 min. Next, the incubated paraffin was stained by DAB and hematoxylin. Finally, the stained paraffin was observed by microscope and the IOD value was used for measuring the expression of VEGF.

### Western blotting

After treatment, HeLa cells were collected, washed and lysed on ice for 30 min in RIPA buffer containing 50 mM Tris (pH 7.4), 150 mM NaCl, 1 % NP-40, 0.5 % sodium deoxycholate, 0.1 % SDS and protease inhibitors (1 mM PMSF); tumors were cut up in RIPA buffer and lysed on ice for 30 mins. Then, after centrifugation at 12000 rpm at 4°C for 10 min, protein concentrations were determined with the BCA protein assay kit. Fifteen micrograms of total cellular protein was separated on 6-12% SDS–PAGE gels and transferred onto a PVDF membrane. The membrane was then blocked in 5 % skim milk in PBST for 1.5 h at room temperature and incubated at 4°C with the primary antibodies overnight. After incubating with anti-rabbit IgG-conjugated HRP at room temperature for 1.5 h, the specific proteins in the blots were detected using ECL chemiluminescence.

### Metabolism of CAPE and CAPE-*p*NO_2_ in HeLa cells

Hela cells were treated with CAPE and CAPE-*p*NO_2_ (20 μM) for 48 h. After treatment, the culture medium was collected, and the HeLa cells were washed with PBS three times. Then, the cells were treated with cold methanol and collected into a centrifuge tube. Equal volumes of chloroform and water (methanol: trichloromethane: water is 1:1:1) were added into the centrifuge tube. After cells were broken, they were centrifuged at 14,000 rpm for 5 min at 4°C, and the upper layer and lower layer of liquid was collected, freeze dried and saved at -80°C.

The precipitate from the culture medium was removed by centrifugation, and the clear supernatant extract was analyzed for metabolites. The chromatographic separation was performed on a Kinetex Core Shell C-18 column (2.1 mm×100 mm, 2.6 μm, Phenomenex, Torrance, CA). Mobile phase A was water-formic acid (1000:1, v/v), and mobile phase B was acetonitrile. The following binary gradient with linear interpolation was used: 0-1 min, 10 % B; 1-5 min, 10 %-80 % B; 5-7 min, 80 % B; 7-7.01 min, 80 %-10 % B; and 7.01-10 min, 10 % B. The flow rate was 300 μL/min, and the column temperature was kept at 30°C.

The LC-QTOF-MS/MS analysis was carried out using a Triple TOFTM 4600 system with a Duo Spray source in the positive and negative electrospray ion mode (AB SCIEX, Foster City, CA, USA). For QTOF-MS, the parameters were optimized as follows: the ion source temperature was 600°C, the mass ranges were set at m/z 50-300, the nebulizer gas was 55 Pa, the heater gas was 55 Pa, the ion spray voltage was 5000 V, the collision energy (CE) was set at 35 eV, and the collision energy spread (CES) was 35 ± 15 eV for TOF MS/MS.

The acquisition and analysis of the LC-MS data were controlled by Analyst^®^ TF 1.6 software and PeakView^®^ 1.2 software (AB SCIEX, Foster City, CA, USA).

### Statistical analysis

All data are presented as the mean ± SD, and experiments were repeated at least three times. Statistical analysis was carried out with one-way ANOVA and Student’s t-test by SPSS software (Version 16.0). ^*^P < 0.05, ^**^p < 0.01: CAPE and CAPE-*p*NO_2_ compared with the control. ^#^*P* < 0.05, ^##^*p* < 0.01: CAPE-*p*NO_2_ compared with CAPE at the same concentration. A P value less than 0.05 was considered statistically significant.

## SUPPLEMENTARY MATERIALS FIGURES


