# Supplementary material for: Inhibited effects of CAPE-pNO2 on cervical carcinoma in vivo and in vitro and its detected metabolites
Source: Oncotarget. 2017 Oct 7;8(55):94197–209. doi: 10.18632/oncotarget.21617 (PMC5706867; doi:10.18632/oncotarget.21617)
Supplement: Supplementary file 1 [file oncotarget-08-94197-s001.pdf]

Inhibited effects of CAPE-*p*NO_2 on cervical carcinoma *in vivo* and *in vitro* and its detected metabolites

SUPPLEMENTARY MATERIALS

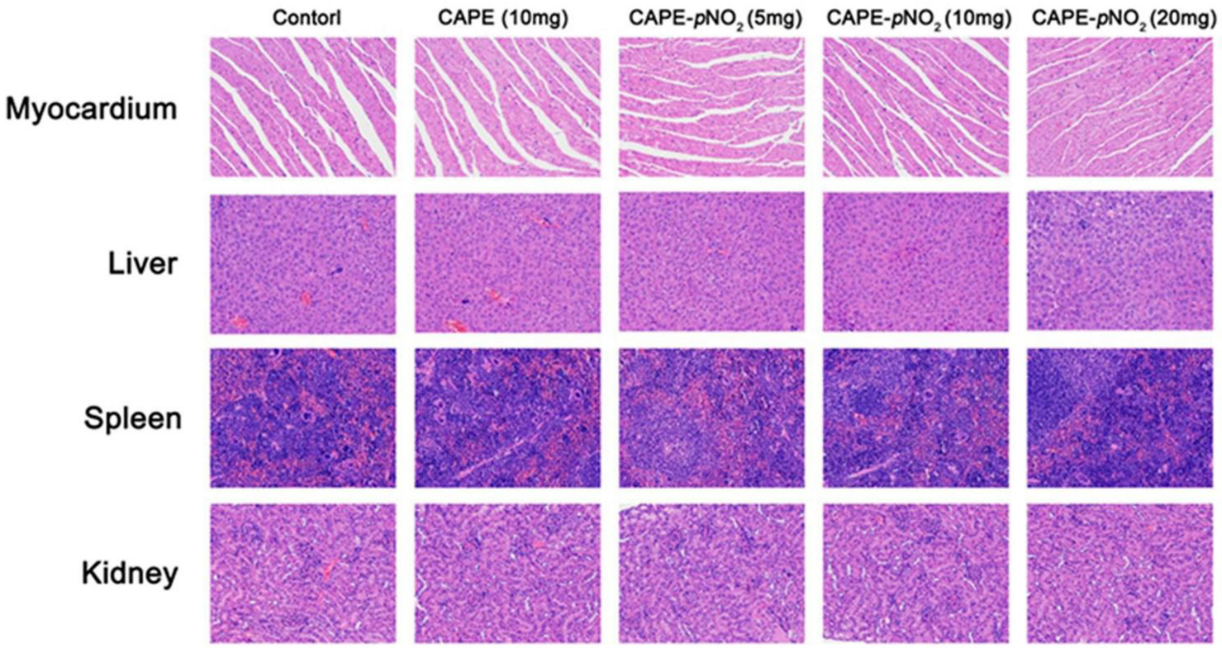

Supplementary Figure 1: Heart, liver, kidney and spleen Paraffin sections of heart, liver, kidney and spleen were stained by hematoxylin and eosin (HE), and there almoet no observable morphological changes after treatments with CAPE and CAPE-*p*NO_2.

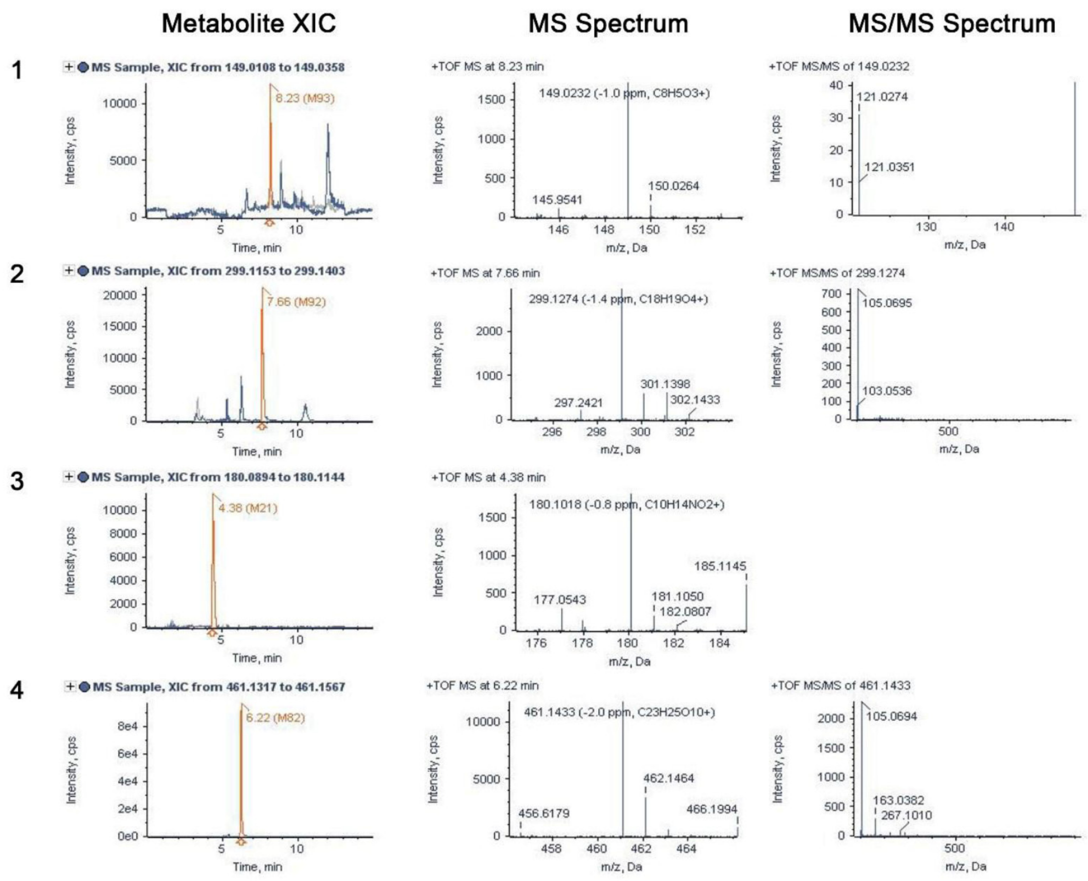

Supplementary Figure 2: Mass spectrum of metabolites of CAPE. The mass spectrum of metabolites of CAPE, and the “1, 2, 3, 4” were correspond to the “1, 2, 3, 4” in Figure 6A.

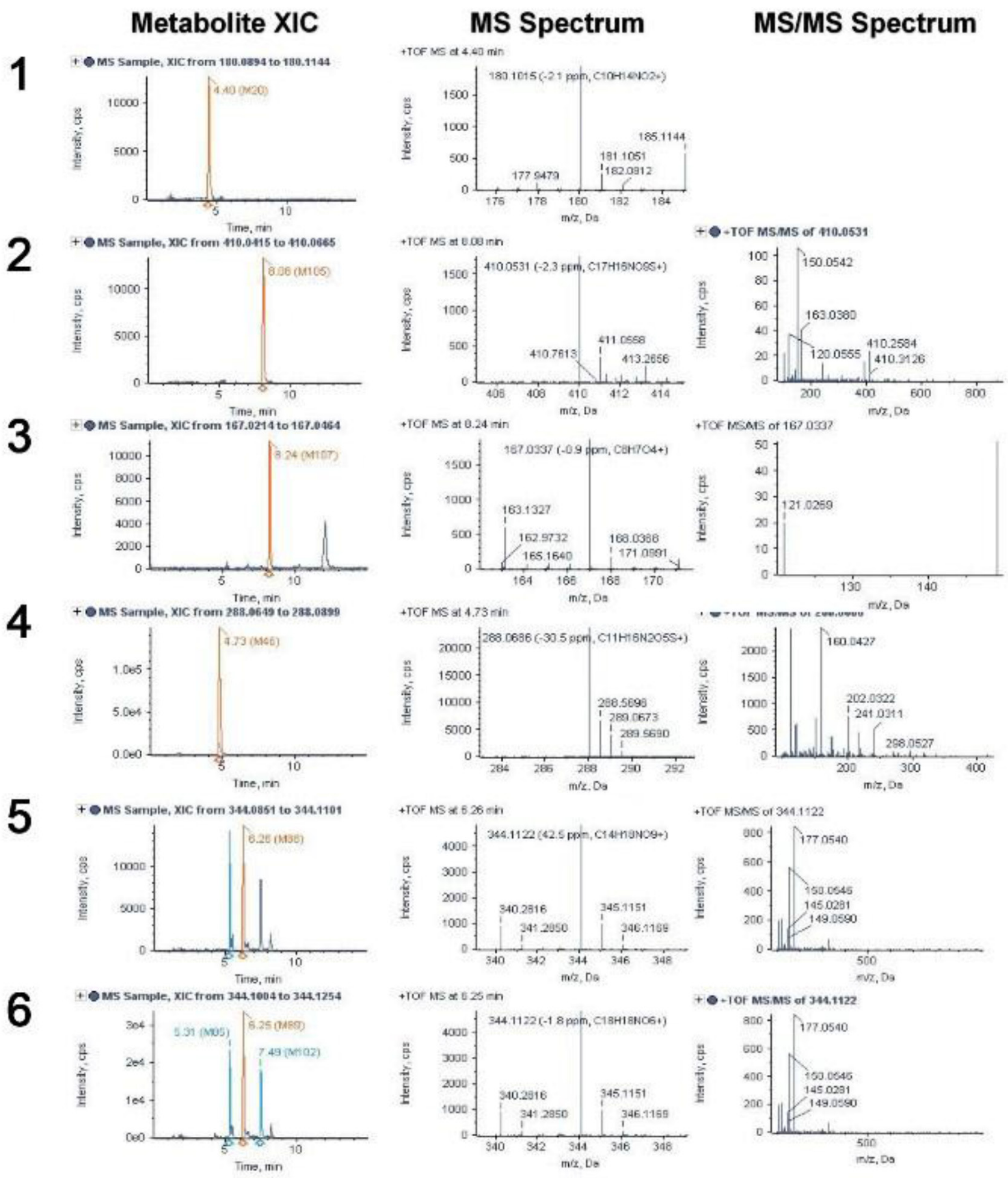

Supplementary Figure 3: Mass spectrum of metabolites of CAPE-*p*NO_2. The mass spectrum of metabolites of CAPE, and the "1, 2, 3, 4, 5, 6" were correspond to the "1, 2, 3, 4, 5, 6" in Figure 6B.
